# CR3 ruffles FcγR’s claim over phagocytic cups

**DOI:** 10.1016/j.jbc.2021.100801

**Published:** 2021-05-19

**Authors:** S.A. Frautschy

**Affiliations:** Geriatric Research Education and Clinical Center, Veterans Greater Los Angeles HealthCare System, Los Angeles, California, USA; Departments of Neurology and Medicine, University of California, Los Angeles, Los Angeles, California, USA

**Keywords:** time-lapse 3D spinning disk confocal microscopy, DAP12, complement receptor 3, filopodia, macrophage, phagocytosis, IgG, IgM, Syk, FcγRs, AD, Alzheimer’s disease, RBCs, red blood cells

## Abstract

Phagocytosis plays diverse roles in biology, but our understanding of the purpose, interplay, and cell signaling mechanisms associated with different modes of phagocytosis is limited, without being able to capture and visualize each step in this rapid process from the beginning to end. A new study by Walbaum *et al.* uses stunning time-lapse 3D imaging of the engulfment of erythrocytes by macrophages *via* sinking, ruffling, and cup formation, unequivocally confirming a visionary 44-year-old theory derived from still electron microscopy photos that phagocytosis mediated by complement receptor CR3 occurs *via* a sinking mechanism and antibody-mediated phagocytosis occurs *via* phagocytic cup formation. The article also challenges the dogma, showing that phagocytic cup formation is not unique to antibody receptor phagocytosis, rather CR3 plays a complex role in different modes of phagocytosis. For example, inhibition of antibody-mediated phagocytosis leads to a compensatory upregulation of CR3-mediated sinking phagocytosis. These findings animate, in vivid colors, processes previously only captured as stills, exposing interactions between different phagocytic mechanisms and altering our basic understanding of this important process.

Phagocytosis continues to intrigue researchers after 140 years, including discoveries of its roles in infectious diseases, aging, cancer, diabetes, and neurodegenerative diseases. In many diseases and in aging, RNA-Seq data show that the expression of genes regulating phagocytosis is dysregulated, including TREM2 and DAP12 in Alzheimer’s disease (AD) (1). However, visualizing step-by-step morphological changes associated with engulfment and related mechanisms is difficult with still images from a process where critical steps last a few seconds and cells return to normal within a few minutes. Now high-resolution video in real time can help us visualize the entire process from the beginning to end, allowing us to better understand the signaling that controls steps in these pathways. Two of the most well-understood phagocytic pathways are complement-mediated phagocytosis and antibody-mediated phagocytosis. The theory proposed by Kaplan in 1977 (2), which has stood up well to time, used elegant scanning and transmission electron microscopy to show that complement-mediated phagocytosis occurs *via* sinking of the cargo into the membrane, whereas antibody-mediated phagocytosis induced the formation of phagocytic cups that rise from the membrane and surround the cargo (Fig. 1*A*). One caveat to this dichotomous theory was the observation that membrane ruffles resembling small incomplete cups were occasionally observed in complement-mediated phagocytosis (2, 3, 4, 5). Ruffles remain a mysterious phenomenon of unknown significance. Walbaum *et al.* reignite interest in this topic, including ruffles, by creating new assays for complement *versus* antibody or dual complement and antibody modes of phagocytosis that facilitate comparison of the associated molecular mechanisms in real time. These efforts not only validate long-held beliefs but also reveal unexpected roles of CR3 in cup formation and synergism between different phagocytic mechanisms involving standard cup formation, ruffles, and sinking (6).

**Figure 1:**
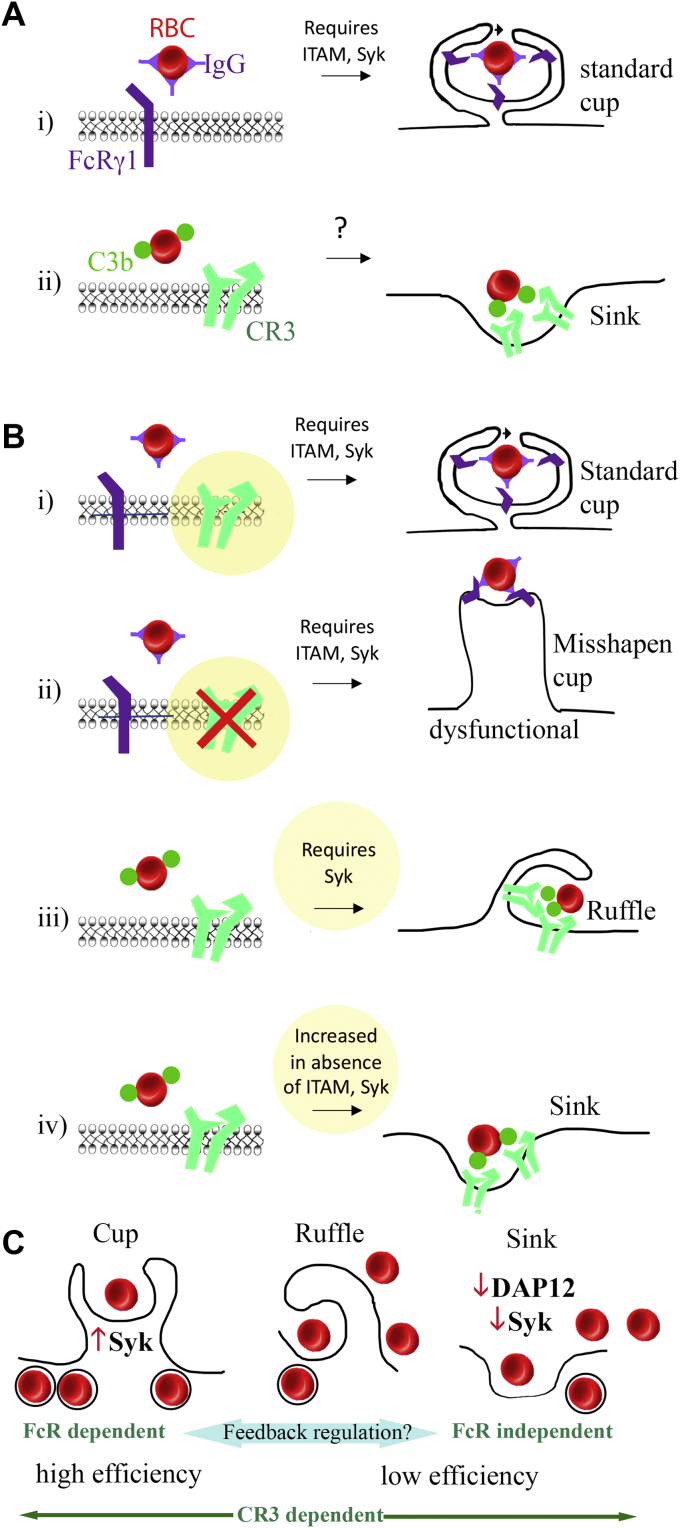
**Time-lapse spinning disk laser confocal microscopy allows a deep dive into elucidating the enigmatic role of CR3 as a master regulator of different modes of phagocytosis.** We now have a better understanding of the role of Syk and CR3 in different modes of phagocytosis. *A*, the schematic reflects early findings with electron microscopy, suggesting that IgG antibody-mediated phagocytosis of RBCs occurred by cup formation, involving ITAM activation of Syk (i), whereas complement-mediated phagocytosis occurred *via* sinking (ii). *B*, live imaging indisputably upholds this theory, showing the fate of each RBC from the beginning to end, but new findings were revealed (shown in *yellow*, i–iv). FcR-mediated cup formation involves CR3 (i) and becomes misshapen and dysfunctional in the absence of CR3 (ii). Unexpectedly, even in the absence of FcR, complement-mediated phagocytosis can be associated with both cup formation (not shown) and ruffles (iii). Although ruffles were previously seen with complement-mediated phagocytosis, this is the first time it was observed that some eventually form functional cups. The lack of ITAM and Syk, which are both necessary for cup formation, drives sinking of RBCs into the membrane (iv). *C*, phagocytic efficiency of sinking or ruffles is typically 50% below that of cup formation, and suppressing signaling mechanisms in cup formation can cause compensatory increase in sinking. These findings raise new questions about the mechanistic interplay between different modes of phagocytosis and their efficiencies and other immunoreceptor signaling pathways (*e.g.*, TLR4 and TREM2). RBC, red blood cell.

Complement-mediated phagocytosis begins when cargo such as red blood cells (RBCs) are bound by complement proteins such as C3b (to make them more recognizable by immune receptors, called ‘opsonization’). These opsonized particles then bind to complement receptors (such as CR3), and downstream signaling is thought to lead to the actin-mediated sinking of the cargo into the membrane. On the other hand, antibody-mediated phagocytosis is initiated upon binding of cargo opsonized by antibodies (such as immunoglobulin G [IgG]) to receptors that recognize the crystallizable fragment of those antibodies (Fcγ receptors). Subsequent signaling from Fcγ receptors, primarily activating ITAM domains and the downstream signal transducer tyrosine kinase Syk, leads to the formation of phagocytic cups that engulf cargo (7). However, there is a lack of visual confirmation of the exact sequence of steps from the beginning to end in the different modes of phagocytosis in a process that takes only a few minutes before RBCs are lysed and macrophages return to normal morphology. This makes it difficult to understand rapid dynamic mechanisms in these distinct and interactive phagocytic pathways.

Walbaum *et al.* developed assays from scratch for different modes of phagocytosis of human RBCs by mouse peritoneal macrophages. Using these assays, they could also study signaling mechanisms by comparing WT with genetically modified mice (KOs of the receptors FcγR or CR3, or of ITAM domains or entire ITAM-bearing adaptor proteins). The assay development included not only ensuring that no nonopsonized RBCs were lysed but also controlling the time course, so each step could be captured using 3D time-lapse imaging for the different modes of phagocytosis explored. These included FcγR-mediated opsonization of RBC by IgG, dual opsonization with IgG and C3b, and complement-mediated phagocytosis (dual opsonization with immunoglobulin M and C3b). Depending on the experiment, this required varying the temperature or depleting C5 from the media to prevent nonphagocytic lysis. This allowed Walbaum *et al.* to visualize the sequence of events in the macrophage membrane throughout RBC uptake and resolution to baseline morphology. These data specifically demonstrate that in the absence of FcγR signaling *via* deletion of downstream ITAM adaptors or Syk, macrophages can no longer ingest IgG-opsonized RBCs. However, when exposed to C3b-opsonized RBCs in the absence of those same ITAM-bearing adapter proteins downstream of antibody-mediated cup formation, cups could still form (likely mediated by a separate ITAM-adaptor protein, DAP12), although the cups were more ruffled in appearance. These findings demonstrate that contrary to the dogma, CR3 does contribute to cup formation (Fig. 1*B*). Consistent with the electron microscopy photos of Kaplan, 3D time-lapse imaging showed FcγR-mediated phagocytic cups could emerge from small areas of contact, such as filopodial (finger-like) extensions. Thus, unlike previously thought, CR3-mediated membrane ruffles (3, 4, 5) could actually evolve into cups.

This surprising revelation that CR3 may modulate *bona fide* cups was further supported by work in CR3 KO cells, which, while capable of ingesting IgG-opsonized RBCs, exhibited peculiar malformations in their phagocytic cups (Fig. 1*B*). The FcγR-mediated phagocytic cups, although initially spherical, became markedly elongated and cylindrical when in contact with IgG-opsonized RBCs—just imagine an amoeba morphing into a slug (Video S15 is a must-see). In addition, after deletion of Syk, absolutely no cups could form and complement-mediated sinking phagocytosis accelerated, as captured in striking videos of RBCs squeezing through narrow orifices in the membrane, contorting into hourglass shapes, and then entering the cell as spherical phagosomes. Unexpectedly, deletion of DAP12 or Syk also accelerated engulfment and increased sinking phagocytic efficiency, apparently to compensate for the complete abolishment of cup formation. This provides solid new evidence of interplay between different modes of phagocytosis.

Overall, these 3D imaging data illustrate the basis for a new model that highlights crosstalk between the CR3- and FcγR-mediated phagocytic clearance pathways (Fig. 1*C*). In the pure IgG antibody-mediated high-efficiency phagocytosis model (in the absence of C3b), the ingestion of IgG-opsonized RBCs is exclusively mediated by phagocytic cup formation (as opposed to sinking), requiring a functional ITAM domain in the FcγR. Nevertheless, and surprisingly, it still requires CR3 for functional cup formation. Even with dual opsonization with both IgG and C3b, RBCs are still phagocytosed with high efficiency, but sinking also emerges. Sinking is further accelerated by the deletion of the FcγR ITAM adaptor or its signal transducer Syk, suggesting crosstalk between the IgG antibody and complement receptors. Even bypassing the FcγR entirely, to examine pure complement receptor-mediated phagocytic cup formation (immunoglobulin M + C3b), still permits some cup formation *via* activation of Syk by either of the two ITAM adaptors (DAP12 and FcR γ-chain). This adds further new evidence for a CR3 role in cup formation, although, as previously described, sinking dominates in the complement pathway (2).

The findings presented by these authors are likely to impact many fields. For example, in AD, phagocytic dysfunction has been revealed to be a major player in pathogenesis and disease progression. CR3 can have important constitutive roles in the clearance of protein aggregates, including beta-amyloid and tau tangles (8, 9). This synergy is also relevant to AD therapeutics; for example, the mechanism of rapid clearance after passive or active beta-amyloid immunization is still not well understood, but based on this new study, is likely to involve phagocytic cup formation. Future studies will also certainly explore the roles of these and other immunoreceptors in different modes of phagocytosis. For example, TLR4 receptors are known to be upregulated by CR3-mediated signaling (10) and also to cause membrane ruffling (3). Furthermore, TREM2 (a microglial receptor that also signals *via* DAP12) is a master regulator of phagocytosis (1) that plays pivotal roles in AD, but its impact on complement-mediated phagocytic cup formation is unknown. This study is likely to further spark the scientific community’s obsession with phagocytosis, while bringing Kaplan’s elegant still photos to life.

## Conflict of interest

The author declares that she has no conflicts of interest with the contents of this article.
